# Circulating CD4+CD161+ T Lymphocytes Are Increased in Seropositive Arthralgia Patients but Decreased in Patients with Newly Diagnosed Rheumatoid Arthritis 

**DOI:** 10.1371/journal.pone.0079370

**Published:** 2013-11-01

**Authors:** Paulina Chalan, Bart-Jan Kroesen, Kornelis S. M. van der Geest, Minke G. Huitema, Wayel H. Abdulahad, Johan Bijzet, Elisabeth Brouwer, Annemieke M. H. Boots

**Affiliations:** 1 Department of Rheumatology and Clinical Immunology, University Medical Center Groningen, Groningen, The Netherlands; 2 Department of Laboratory Medicine, University Medical Center Groningen, Groningen, The Netherlands; 3 Groningen research initiative on healthy ageing and immune longevity (GRAIL), Groningen, The Netherlands; Karolinska Institutet, Sweden

## Abstract

Improved understanding of the immune events discriminating between seropositive arthralgia and clinical synovitis is of key importance in rheumatology research. Ample evidence suggests a role for Th17 cells in rheumatoid arthritis. We hypothesized that CD4+CD161+ cells representing Th17 lineage cells may be modulated prior to or after development of clinical synovitis. Therefore, in a cross-sectional study, we investigated the occurrence of CD4+CD161+ T-cells in seropositive arthralgia patients who are at risk for developing rheumatoid arthritis and in newly diagnosed rheumatoid arthritis patients. In a prospective study, we evaluated the effect of methotrexate treatment on circulating CD4+CD161+ T-cells. Next, we assessed if these cells can be detected at the level of the RA joints. Precursor Th17 lineage cells bearing CD161 were found to be increased in seropositive arthralgia patients. In contrast, circulating CD4+CD161+T-cells were decreased in newly diagnosed rheumatoid arthritis patients. The decrease in CD4+CD161+ T-cells correlated inversely with C-reactive protein and with the 66 swollen joint count. Methotrexate treatment led to normalization of CD4+CD161+ T-cells and reduced disease activity. CD4+CD161+ T cells were readily detected in synovial tissues from both early and late-stage rheumatoid arthritis. In addition, synovial fluid from late-stage disease was found to be enriched for CD4+CD161+ T-cells. Notably, synovial fluid accumulated CD4+CD161+T-cells showed skewing towards the Th1 phenotype as evidenced by increased interferon-γ expression. The changes in peripheral numbers of CD4+CD161+ T-cells in seropositive arthralgia and early rheumatoid arthritis and the enrichment of these cells at the level of the joint predict a role for CD4+CD161+ T-cells in the early immune events leading to clinical synovitis. Our findings may add to the development of RA prediction models and provide opportunities for early intervention.

## Introduction

Rheumatoid arthritis (RA) is a chronic autoimmune disease characterized by joint inflammation of synovial tissue eventually leading to cartilage and bone destruction. Early and combination treatment strategies were shown to be effective in controlling joint inflammation and prevention of bone erosions [1]. Thus, early recognition of RA and identification of individuals at risk for arthritis development would open opportunities for early clinical intervention. 

Autoantibodies such as rheumatoid factor (RF) and anti-cyclic citrullinated peptide antibodies (ACPA or anti-CCP) can be found in individuals already years before clinical synovitis becomes manifest. In a prospective study design, arthritis development in arthralgia patients was found to be associated with anti-CCP status [2,3]. Of this seropositive arthralgia patient (SAP) group, 35% developed arthritis after a median follow-up period of 12 months [3]. The majority of these patients (92%) fulfilled the 2010 American College of Rheumatology (ACR) criteria for classification of RA. 

Understanding the immune events involved in the transition to clinical synovitis, and translation of these insights into the development of biomarkers is key [4]. Ample evidence suggests a role for Th17 cells in the development of RA [5]. In recent years, evidence was provided for the involvement of Th17 cells in the initiation phase of RA. First, the development of a cytokine environment favoring Th17 generation is an early event in RA pathogenesis [6]. Second, in line with this, pre RA patients were found to show increased serum levels of IL-17 prior to the manifestation of clinical synovitis but these levels dropped significantly following the transition to RA [7]. Although Th17 cells were detected in rheumatoid synovial tissue, Th17 cells appear to be outnumbered by interferon (IFN)-γ-producing Th1 cells at the level of the inflamed joint [8,9]. The latter may be explained by reprogramming of Th17 cells to Th1 cells by synovial fluid derived IL-12, as shown in juvenile idiopathic arthritis [10,11]. 

Human CD161 is the homologue of mouse NK cell receptor-P1A and constitutes a type II transmembrane glycoprotein with characteristics of the C-type lectin superfamily [12]. CD161 is now considered a marker of Th17 lineage cells [13-15]. All human IL-17 producing cells are thought to originate from CD161+ precursors in umbilical cord blood and newborn thymus [13]. CD161 expression is induced by RAR-related orphan receptor C (RORC), the Th17 lineage transcription factor [16]. It seems that CD161 expression is maintained throughout the life cycle of the cell since it is detectable on memory circulating and tissue infiltrating Th17, Th17/Th1 and Th1 lymphocytes. CD161 expression thus presents a means to determine Th17 ancestry in peripheral and tissue infiltrating CD4+ T-cells [17,18]. 

We hypothesized that CD4+CD161+ T-cells representing Th17 lineage cells are involved in the initiation phases of RA and that peripheral numbers of these cells may be modulated prior to or after the development of clinical synovitis. Therefore, in a cross-sectional study, we investigated the occurrence of CD4+ CD161+ T-cells in SAP, newly diagnosed, treatment-naïve RA patients at baseline and at 3 and 6 months after start of methotrexate (MTX) treatment. Next, we assessed if CD4+CD161+ T-cells can be detected in early and late-stage RA synovial tissue. In late-stage RA, paired samples of peripheral blood and synovial fluid were studied. Our study revealed altered numbers of peripheral CD4+CD161+ T-cells in SAP versus early RA patients and a relative enrichment of these cells in RA joints. The data suggest a role for CD4+CD161+ T-cells in the early immune events leading to clinical synovitis.

## Materials and Methods

### Ethics statement

Rheumatoid arthritis (RA) and seropositive arthralgia patients (SAP) gave their written informed consent. Twentyfour healthy control (HC) volunteers were recruited in 2010 and 2011 for establishing reference values on leukocyte subsets and T cell cytokine production. Documented oral (n = 20) or written informed consent (n = 4) was obtained. Documented oral consent and anonymisation of HC blood samples by a registered medical immunologist was approved by the Institutional Review Board. At time of blood sampling, healthy volunteer donors were requested to fill out a health questionnaire. All procedures were in accordance with institutional guidelines and approved by the local medical ethics committee (UMCG Groningen, the Netherlands). METC numbers: 2007.148, 2009.118, 2011.306 and 2012.375.

### Study Populations

Starting February 2012, twenty-six patients with a positive anti-CCP and/or IgM-rheumatoid factor (RF) status and (a history of) arthralgia, but not arthritis, defined as SAP, were recruited at the rheumatology outpatient clinic at the UMCG. Absence of arthritis was confirmed by physical examination of 44 joints by a trained medical doctor and a senior rheumatologist. 

Thirty-five newly diagnosed, treatment-naive RA patients were recruited starting in 2010. All patients fulfilled the ACR 1987 or 2010 criteria [19,20] with a mean duration of preceding symptoms of 10,2 ± 16,5 months. Twenty-six and 12 RA patients were followed prospectively for a period of 3 (14 ± 3 weeks) and 6 months (29 ±7 weeks) respectively after start of MTX treatment. 

Paired samples of peripheral blood (PB) and synovial fluid (SF) were obtained from 11 RA patients with late-stage RA. All of these patients fulfilled the ACR 1987 criteria and were treated with different disease modifying anti-rheumatic drugs (DMARDs, e.g. MTX, Etanercept, Adalimumab and Prednisolone).

Twenty-four healthy subjects, matched for age, sex and ethnicity, were included as a control group. Exclusion criteria were inflammation, malignancy (past or present), or use of immune suppressive drugs. 

Demographic and clinical characteristics of patients and controls are summarized in Table 1. In addition, synovial tissues (ST) were obtained from 15 early, treatment naïve RA patients undergoing knee or ankle arthroscopy. Four out of 15 biopsy tissues contained clear cellular infiltrates and were processed for immunohistochemistry (IHC, n = 4. Three out of 4 were female, median age of 60.5 (range 53,2 - 67,8), 3 out of 4 were anti-CCP positive and all 4 were RF positive). In addition, we obtained ST biopsies from 4 RA patients with late-stage, active RA (All 4 patients were female with a mean age of 54,0± 4,4; C-reactive protein (CRP) median value (range) of 24,0 mg/L (7,0-63,0); erythrocyte sedimentation rate (ESR) median value (range) of 48,5 mm/h (19,0-51,0); 1 out of 4 was anti-CCP-positive; 3 out of 4 were RF-positive).

**Table 1:** Demographic and clinical characteristics of subjects included in the study.

			**Longitudinal study**			
	**HC**	**SAP**	**Early RA**	**Early RA**	**Early RA**	**Late-**
			**Baseline**	**3 months**	**6 months**	**Stage RA**
				**MTX**	**MTX**	
N	24	26	35	26	12	11
Age [yrs]	58.8 ±10.4	55.2 ±10.5	59.9 ±10.9	59.7 ±11.5	56.9 ±9.7	62.1 ±12.5
mean ± SD						
Gender (% female)	79.2	65.4	80.0	77.0	42.0	63.6
SE (% positive)	66.7	66.7	70.0	81.2	100	nd
Disease duration [yrs]	na	na	na	na	na	5.8
median (range)						(2.4-43.9)
Erosive (%) X ray	na	na	20.0	nd	33.3	63.6
CRP [mg/L]	na	5.0	14.0	5.0	5.0	21.5
median (range)		(5-29)	(5-118) *	(5-45) †	(5-16) †	(5-49) * #
ESR [mm/h]	na	14.0	26.0	18.0	12.0	23.5
median (range)		(2-69)	(2-96) *	(5-76)	(4-55)	(3-80)
RF (% positive)	na	73.1	74.3	76.9	83.3	63.6
Anti-CCP (% positive)	na	96.0	68.6	73.1	75.0	90.9
DAS28 (mean ± SD)	na	na	5.1 ±1.3	3.7 ±1.3 †	2.8 ±1.0 †	2.4 ±1.0 †

SE = shared epitope (SE-containing alleles are HLA-DRB1*0401, * 0404, * 0405, * 0408, * 0101, * 0102 and * 1001); CRP= C-reactive protein; ESR= erythrocyte sedimentation rate; RF= rheumatoid factor (positive score defined as ≥ 15 IU/mL); Anti-CCP= anti- cyclic citrullinated peptides antibodies (positive score defined as > 10 IU/mL); DAS28= disease activity score 28; nd = not determined; na = not applicable. * p < 0.05 compared to seropositive arthralgia patients; † p < 0.05 compared to RA patients at baseline; # p < 0.05 compared to RA patients after 3 mo MTX treatment. Seropositive arthralgia patients (SAP) inclusion criteria were anti-CCP and /or RF seropositivity and arthralgia affecting at least one joint.

### Anti-CCP and RF Determination

Anti-CCP serum levels were determined by anti-IgG CCP fluorescent enzyme immuno assay on Phadia 250 (Thermo Fisher Scientific, Uppsala, Sweden). RF total Ig serum levels were determined by turbidimetry using modular analyzer (Roche, Mannheim, Germany). Seropositive status was defined as anti-CCP serum levels of ≥ 10 IU/mL and/or RF serum levels of ≥ 15 IU/mL. 

### Flowcytometric Analysis of T-cell-surface Markers and Cytokine Expression

The absolute numbers and the phenotype of lymphocytes in PB and SF were determined using multicolor flowcytometry. Briefly, 100 µl of EDTA anti-coagulated PB or SF was collected and stained, according to the manufacturer’s instructions, with combinations of the following antibodies: anti-CD4 PerCP, anti-CD8 PerCP, anti-CD45RO FITC, anti-CCR7 PE, anti-CD161 APC (BD Biosciences, San Jose, CA, USA) or anti-CD3 eFluor 605NC, anti-CD4 eFluor450 (eBioscience, San Diego, CA, USA), CD8 PerCP, anti-CD45RO FITC, anti-CCR7 PE-Cy7 (BD Biosciences), anti-CD161 PE ((Miltenyi Biotec, Leiden, The Netherlands). 

Absolute numbers of leucocytes, CD3 and CD4 T-cells were determined using the BD MultiTest TruCount method with six-color MultiTest reagents detecting CD45, CD3, CD4, CD8, CD19, CD16+CD56 (BD) using a lyse-no-wash preparation method as described by the manufacturer. Flowcytometry was performed on FACSCanto II (BD) and analysis was performed using FACSCanto Clinical Software (BD). CD4+ T-cells were further characterized by analyzing expression of CCR7 and CD45RO [21]. Percentages of T lymphocytes with a naïve (T_Naive_), central memory (T_CM_), effector memory (T_EM_) and terminally differentiated (T_EMRA_) phenotype within the total CD4 counts were used to calculate the absolute numbers of these subsets.

To assess the potential of CD4+ CD161+ T-cells to produce cytokines, 100 µl of heparinized PB or 100 µl of SF was stimulated with phorbol myristate acetate (PMA) (50 ng/mL) and ionomycin (1,6 µg/mL) in the presence of brefeldin A (BFA) (10 µg/mL) (Sigma Aldrich, Zwijndrecht, The Netherlands) for 4 h at 37°C. After stimulation, erythrocytes were lysed with ice-cold ammonium chloride buffer (pH 7,4 for 10 min on ice). Cells were fixed and permeabilized using Fix & Perm Cell Permeabilization Reagents (Life Technologies, Bleiswijk, The Netherlands) and stained with anti- IFNγ PerCP-Cy5.5, anti-tumor necrosis factor (TNF)-α PerCP-Cy5.5 (BioLegend, San Diego, CA, USA), anti-CD3 eFluor605NC, anti-CD4 eFluor450, anti-IL-17 FITC, Perforin FITC (eBioscience), anti-CD8 APC-H7 (BD) and anti-CD161 APC (Miltenyi Biotec). Mouse isotype controls were used in parallel at the same concentrations. Cells were analyzed using FACS Calibur and LSR II flowcytometers (BD Biosciences), and data analysis was performed with FlowJo™ software (Tree Star, Ashland, OR, USA).

### Synovial Tissue and IHC

ST biopsies, obtained from 15 newly diagnosed, treatment-naïve RA patients undergoing knee or ankle arthroscopy were snap frozen and stored at -80 °C. Biopsy sections were first analysed for the presence of cellular infiltrates. Clear infiltrates were detected in 4 out of 15 patient biopsies. Positive biopsies were examined for the presence of CD161-expressing T cells. Cryostat sections (5 µm) were cut and stored at -20 °C. Air-dried sections (30 min) were fixed with 3% paraformaldehyde and 0.3% glutaraldehyde for 10 min. Sections were incubated with 0.1% hydrogen peroxide (Merck, Amsterdam, The Netherlands) for 10 min or Avidin/Biotin Blocking kit (Vector, Burlingame, CA, USA) according to the manufacturer’s instructions. Following washing, sections were incubated with the primary antibodies: anti-CD161 (polyclonal rabbit anti-human CD161 from Sigma-Aldrich) diluted 1:20, anti-CD3 (monoclonal mouse anti-human CD3 from Dako, Glostrup, Denmark,) diluted 1:50, anti-CD4 (monoclonal mouse anti-CD4 from Lifespan BioSciences, Seattle, WA, USA) diluted 1:10 in PBS for 1 hr at RT. Following washing, secondary donkey anti-rabbit-alkaline phosphatase (AP) antibody diluted 1:200, goat anti-mouse IgG1-biotin antibody diluted 1:100 and goat anti-mouse IgG2a-HRP antibody diluted 1:200 (SouthernBiotech, Birmingham, AL, USA) were used respectively. After incubation for 1 hr, CD3 stained tissue sections were incubated with streptavidin-AP (SouthernBiotech) for 30 min at RT. AP substrate (Sigma-Aldrich) or HRP (Dako) were used according to the manufacturer’s instructions. Tissue sections were counterstained with hematoxylin (Merck).

### Synovial Tissue and Cell Isolation

ST biopsies, obtained from late-stage RA patients undergoing hip or knee joint replacement surgery, were rinsed with Dulbecco’s modified Eagles Medium (DMEM, Life Technologies), supplemented with 60 μg/mL gentamycin (Life Technologies) and cut into small fragments using a scalpel. The minced tissue was then incubated with 80 units/mL of hyaluronidase from bovine testes (Sigma-Aldrich) at 37°C. After 15 min. collagenase type IV from *Clostridium histolyticum* (Life Technologies) was added (final concentration of 1 mg/mL). Tissue was further digested, while rotating (225 rpm) for 2 hr at 37°C. After filtration and washing cells were stained with the following antibodies: anti-CD3 eFluor605NC, anti-CD4 eFluor450 (eBioscience), anti-CD19 PerCP, anti-CD161 PE (BD). 

### Statistical Analysis

Power analysis for the sample size calculation was performed based on the results obtained from a pilot experiment involving 20 RA patients and 20 HC. A 2-sided power analysis was done with a confidence interval and power of 95% and 90%, respectively. Results are expressed as mean ± standard deviation (SD) or median (range) for normally distributed and non-normally distributed data, respectively. Normally distributed data was analyzed using ANOVA and unpaired t test. Non-normally distributed data was analyzed using Kruskal-Wallis test and Mann-Whitney 2-tailed test. Correlations between cell numbers and clinical data were evaluated by nonparametric Spearman's correlation analysis. Paired samples analysis was performed with Wilcoxon signed rank test. Generalized estimating equations (GEE) analysis was used to analyze parameters over time within patients. Simple contrasts were used to compare follow-up visits to baseline. Statistical analysis was performed with GraphPad Prism version 5.0 (GraphPad Software, San Diego, CA, USA) and IBM SPSS Statistics 20 (SPSS, Chicago, IL, USA). P<0.05 was considered statistically significant.

## Results

### Circulating Effector Memory Cells are increased in Seropositive Arthralgia Patients when compared to Newly Diagnosed RA

To identify immune markers associated with arthralgia and/or clinical synovitis, we first assessed if the peripheral leukocyte pool was altered in SAP and in newly diagnosed, DMARD-free RA patients (when compared to healthy controls). Absolute numbers of leukocytes, total T-cells and CD4+ T-cells were comparable between groups (Table 2). Next, CD4+ T-lymphocytes with a naïve (T_Naive_), central memory (T_CM_), effector memory (T_EM_) and terminally differentiated effector memory (T_EMRA_) phenotype, defined by expression of CD45RO and CCR7, were assessed. Absolute numbers of T_Naive_, T_CM,_ T_EM_ and T_EMRA_ in SAP were similar to healthy controls. No statistically significant differences were noted. Interestingly, absolute numbers of T_EM_ and T_EMRA_ were significantly elevated in SAP when compared to RA. In addition, absolute CD4+ T_Naive_ counts tended to be increased in SAP and were significantly increased in newly diagnosed RA patients when compared to HC. The latter phenomenon is in line with previous reports on expansion of naïve phenotype cells in RA [22]. 

**Table 2:** Absolute numbers of circulating leukocyte subsets in HC, SAP and RA patients.

	**HC**	**SAP**	**RA Baseline**
N	20	26	30
CD45+			
cell absolute count x106/mL,	1.89	1.91	1.86
median (range)	(1.25-2.63)	(0.89-12.3)	(1.31-3.01)
CD3+			
cell absolute count x106/mL,	1.34	1.42	1.41
median (range)	(0.93-1.80)	(0.51-3.63)	(0.83-2.50)
CD3+CD4+			
cell absolute count x106/mL,	0.96	1.00	0.90
median (range)	(0.49-1.29)	(0.39-2.44)	(0.50-1.68)
CD4+ Naïve (CD45RO-CCR7+)			
cell absolute count x106/mL,	0.25	0.36	0.40 *
median (range)	(0.09-0.58)	(0.07-1.45)	(0.15-0.95)
CD4+ Central Memory (CD45RO+CCR7+)			
cell absolute count x106/mL,	0.28	0.28	0.28
median (range)	(0.03-0.39)	(0.12-0.84)	(0.08-0.72)
CD4+ Effector Memory (CD45RO+CCR7-)			
cell absolute count x106/mL,	0.26	0.31 †	0.25
median (range)	(0.13-0.58)	(0.11-0.53)	(0.11-0.49)
CD4+ Terminally Differentiated EM			
(CD45RO-CCR7-)			
cell absolute count x106/mL,	0.020	0.034 †	0.018
median (range)	(0.004-0.56)	(0.005-0.23)	(0.004-0.14)

* p < 0.05 RA patients at baseline vs HC; † p < 0.05 Seropositive arthralgia patients (SAP) vs RA patients at baseline (Mann-Whitney test). The absolute numbers of CD45+, CD3+, CD3+CD4+ lymphocytes were determined using the BD MultiTest TruCount method. Relative values (%) of T lymphocytes with a naïve (TNaive), central memory (TCM), effector memory (TEM) and terminally differentiated effector memory (TEMRA) phenotype within the total CD4 counts were used to calculate the absolute numbers of these subsets.

### Circulating CD4+CD161+ T lymphocytes are Increased in Seropositive Arthralgia Patients but Decreased in Patients with Newly Diagnosed RA

**Figure 1:**
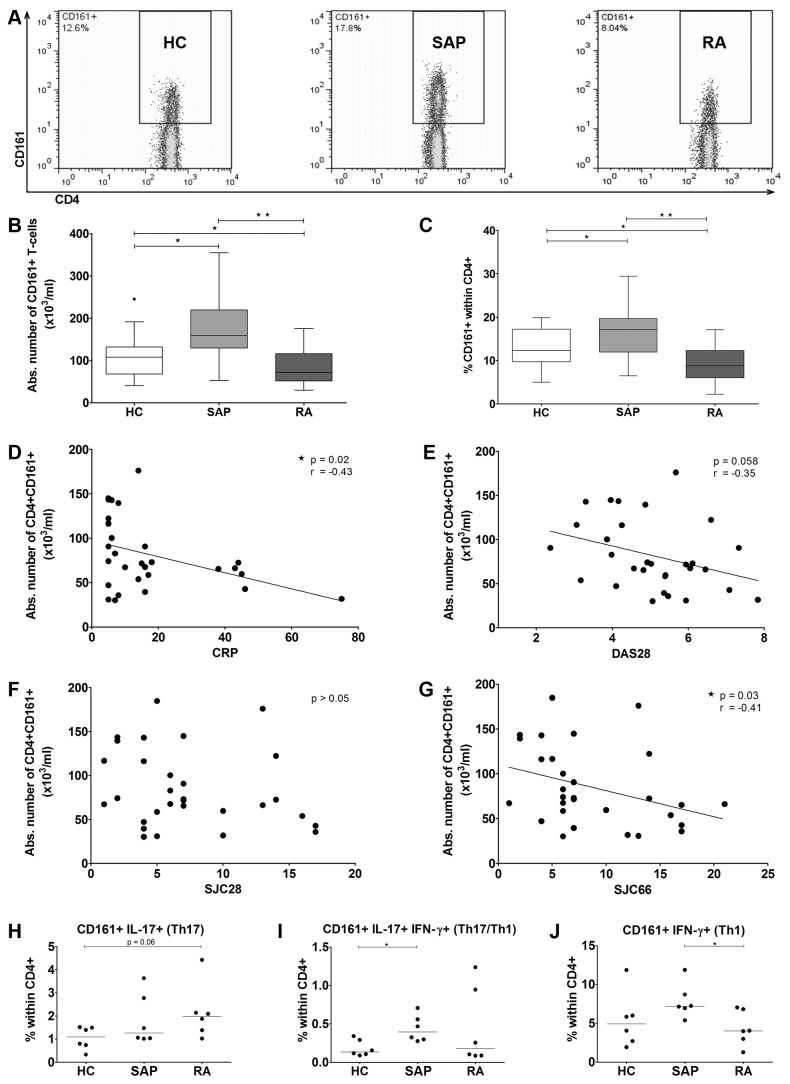
Altered dynamics of circulating CD4+CD161+ T-cells in seropositive arthralgia patients and in newly diagnosed RA patients. (A) Representative dot plots showing proportions of CD4+CD161+ T-cells in the study groups. The absolute number (B) and the frequency (C) of CD161+ cells within CD4+ T-cells from healthy donors (n=20), SAP (n=26) and early RA patients (n=35; Mann-Whitney test). Horizontal line in the box represents the median value. Boxes represent interquartile range and whiskers represent the actual range. Symbols outwith the boxes represent outliers. The correlation between the absolute number of CD161+ cells within CD4+ T-cells and (D) CRP (mg/l), (E) DAS28, (F) SJC28 and (G) SJC66 in RA patients (n=30; Spearman coefficient analysis). Frequency of CD4+CD161+ -cells expressing (H) IL-17, (I) IL-17 and IFN-γ or (J) IFN-γ alone within total CD4+ from HC (n=6), SAP (n=6) and early RA patients (n=6; Mann-Whitney test). Statistical significance is indicated as * p ≤ 0.05, ** p ≤ 0.001, CRP= C-reactive protein, DAS= disease activity score, SJC= swollen joint count.

We next investigated circulating T-cells expressing the Th17 lineage marker CD161 in SAP and RA patients. Whereas absolute numbers of CD4+CD161+ T-cells were found increased in SAP, a significant decrease of these cells was noted in newly diagnosed RA patients (Figure 1A, B). Similar results were obtained when proportions of CD161+ T-cells within the CD4 subset were calculated (Figure 1C). Next, we assessed whether the decrease of the CD4+CD161+ population was associated with clinical measures of disease activity. The absolute numbers of CD4+CD161+ T-cells as seen in newly diagnosed RA correlated inversely with CRP (r = -0,43 and P = 0.02, Figure 1D). Moreover, CD4+CD161+ T-cells tended also to correlate inversely with the disease activity score 28 (DAS28, r = -0,35 and P = 0.058, Figure 1E). Absolute numbers of CD4+CD161+ cells did not correlate with the 28 swollen joint count (SJC) (r = -0,26 and P = 0.18, Figure 1F) in which ankles and feet are not included. Interestingly, absolute numbers of CD4+CD161+ cells were found to correlate inversely with the total 66 SJC (r = -0,41 and P = 0.03, Figure 1G). 

Previously, CD4+CD161+ T-cells were found to contain Th17 (defined by expression of IL-17 but not IFN-γ) and two progeny subsets: Th17/Th1 (defined by expression of both IL-17 and IFN-γ) and Th1 (the so-called non classical Th1 defined by expression of IFN-γ but not IL-17) [17,18]. We assessed the relative frequencies of these cells by analyzing the CD4+CD161+ T-cell cytokine producing potential in the different groups (Figure 1H-J). Frequencies of circulating Th17 cells tended to be increased in newly diagnosed RA when compared to HC (Figure 1H). Th17/Th1 double positive cells were found increased in SAP (Figure 1I). Non classical Th1 were found to be decreased in newly diagnosed RA when compared to the SAP group (Figure 1J).

### Circulating CD4+CD161+ T-cells normalize following Treatment

**Figure 2:**
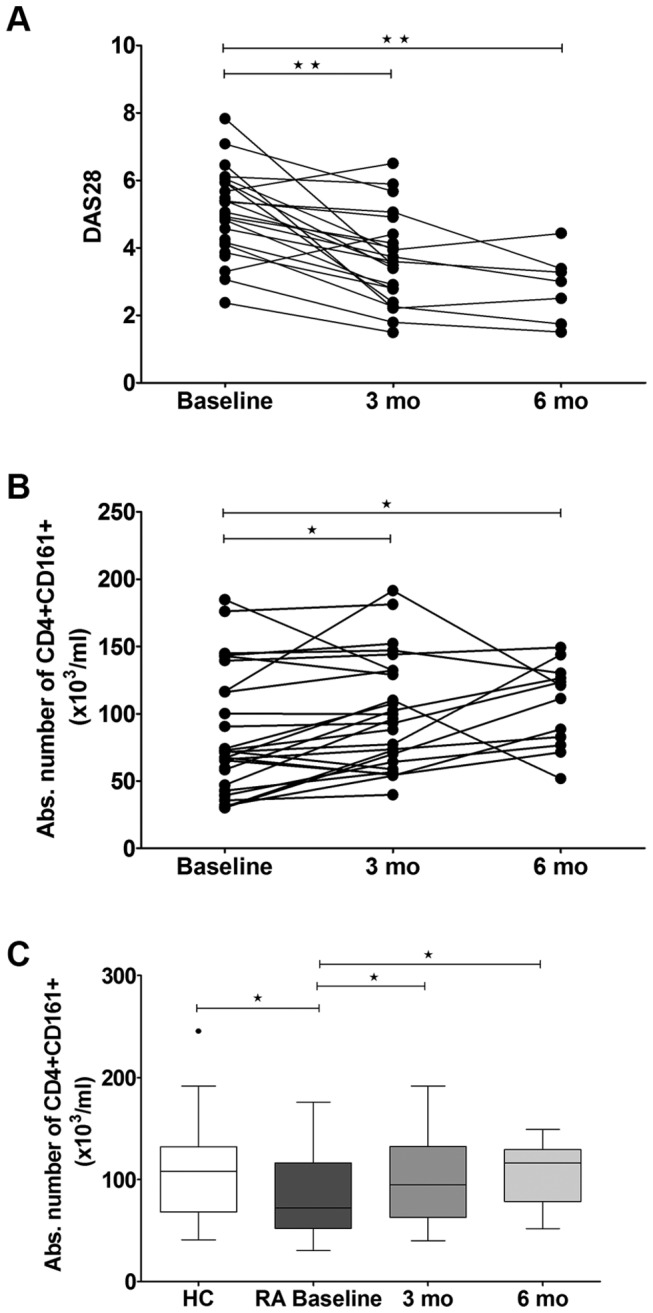
Circulating CD4+CD161+ T-cells normalize following MTX treatment. (A) DAS28 and (B) the absolute number of CD4+CD161+ T-cells from RA patients at baseline (newly diagnosed; n=30), after 3 months (n=22) and 6 months (n=7) of MTX treatment (GEE analysis); (C) Comparison of the absolute number of CD4+CD161+ T-cells between HC (n=20) and RA patients at baseline (n=30; Mann- Whitney test) or RA patients at baseline (n=30) and RA patients after 3 months (n=26) or 6 months (n=12) of MTX treatment (Wilcoxon matched pairs test). Horizontal line in the box represents the median value. Boxes represent interquartile ranges and whiskers represent the actual range. Statistical significance is indicated as * p ≤ 0.05, ** p ≤ 0.001.

Newly diagnosed RA patients were assessed at baseline (before start of MTX treatment) and at 3 and 6 months after start of treatment for absolute numbers of circulating CD4+CD161+ T-cells and for clinical parameters of disease activity. MTX treatment significantly reduced CRP and Disease Activity Score (DAS)28, but not ESR, at 3 and 6 months when compared to baseline (Table 1). Importantly, the reduction in CRP and DAS28 was associated with an increase in the absolute number of circulating CD4+CD161+ T-cells (Figure 2A, B). Notably, the numbers of PB CD4+CD161+ T-cells increased to the level observed in healthy subjects at 3 and 6 months (Figure 2 C). The data merit further study into the utility of circulating CD4+CD161+ T-cells as a potential biomarker of synovitis in RA.

### CD4+CD161+ T-cells are found at Inflamed Sites in RA Joints

**Figure 3:**
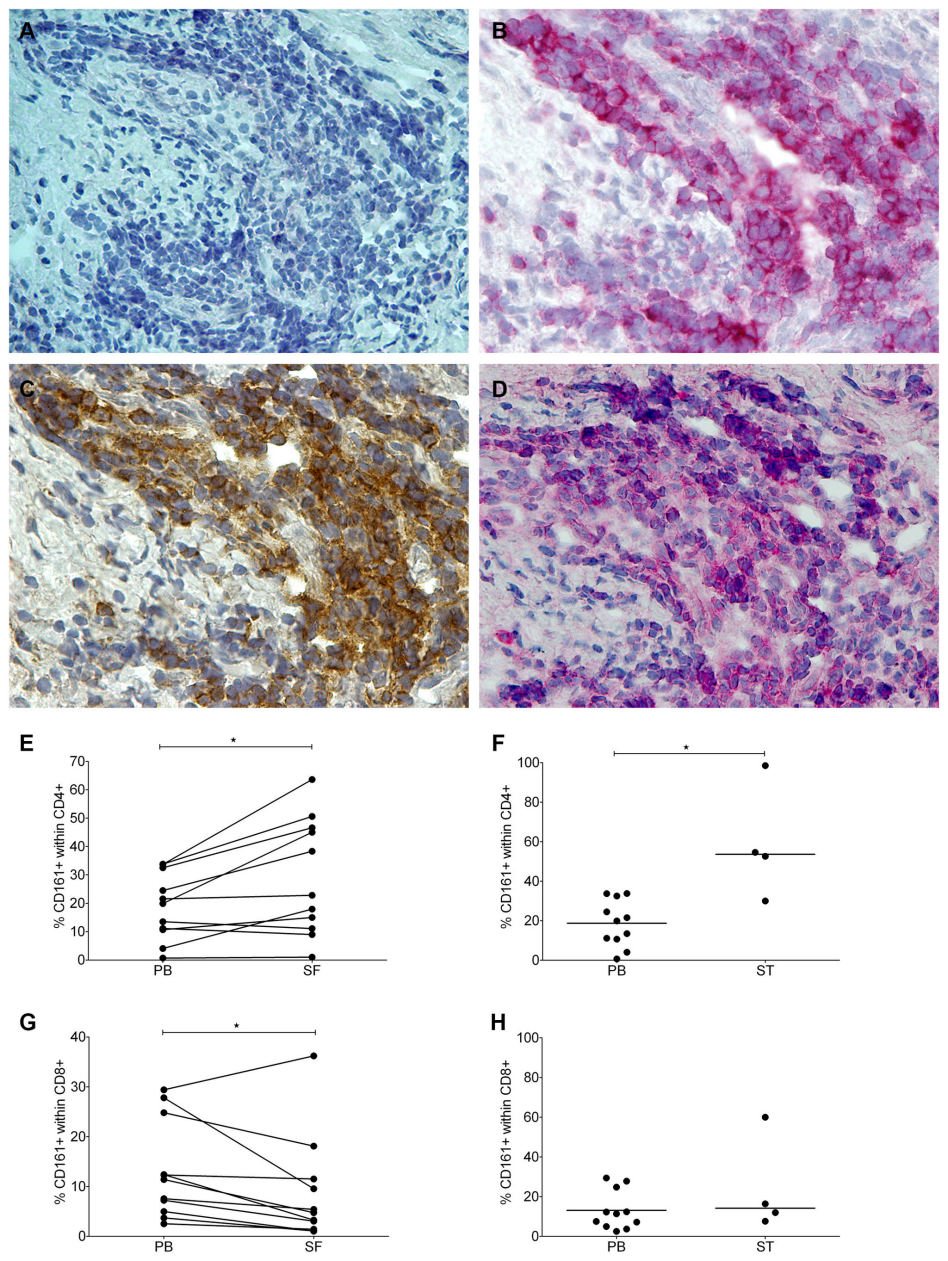
CD4+CD161+ T-cells are readily detected at the level of the joint in newly diagnosed and in late-stage RA. Detection of T cells expressing CD161 in ST obtained from a newly diagnosed RA patient using IHC on consecutive cryostat sections (Krenn score = 4 [37]). A representative example is shown (magnification 40x). Blanc (A), CD3 (B), CD4 (C), CD161 (D). Analysis of the number of CD4+CD161+ T-cells from paired PB and SF and non-paired PB and ST from late-stage RA. Frequency of CD161+ cells within (E, F) CD4+ and (G, H) CD8+ T-cells were compared between paired samples of PB and SF (n=6; Wilcoxon matched pairs test) or PB and enzyme-digested ST (n=4; Mann-Whitney test).

CD161 may function as an adhesion molecule and thereby facilitate migration [14,23]. To assess if the observed decrease of circulating CD4+CD161+ T-cells in newly diagnosed RA patients may be explained by their homing to the site of inflammation, we analyzed CD161 expression in ST samples obtained via arthroscopy in this group. CD4+ CD161+ T-cells were readily detected in ST sections using IHC. Representative staining of consecutive synovial biopsy sections showed clear staining for CD161 in the area’s infiltrated by CD3- and CD4- expressing cells (Figure 3A-D). 

Next, we investigated CD4+CD161+ T-cells in patients with late-stage disease. To that end, relative frequencies of these cells were assessed in paired samples of PB and SF. Also, we assessed the presence of CD4+CD161+ T-cells in digested ST biopsies by flowcytometry. The frequency of CD4+CD161+ T-cells in the SF was significantly increased when compared to PB (mean value 29,2% within the total CD4+ in SF vs. 18,7% within total CD4+ in PB, Figure 3E). Similarly, flowcytometric analysis of digested ST from late-stage RA showed an increased frequency of CD4+CD161+ T-cells (median value 58,9% of total CD4+ in ST, Figure 3F). In contrast, we did not observe an accumulation of CD8+CD161+ T-cells in SF or ST (Figure 3G, H). 

### Synovial Fluid-derived CD4+CD161+ T-cells show enhanced IFN-γ-producing Capacity

**Figure 4:**
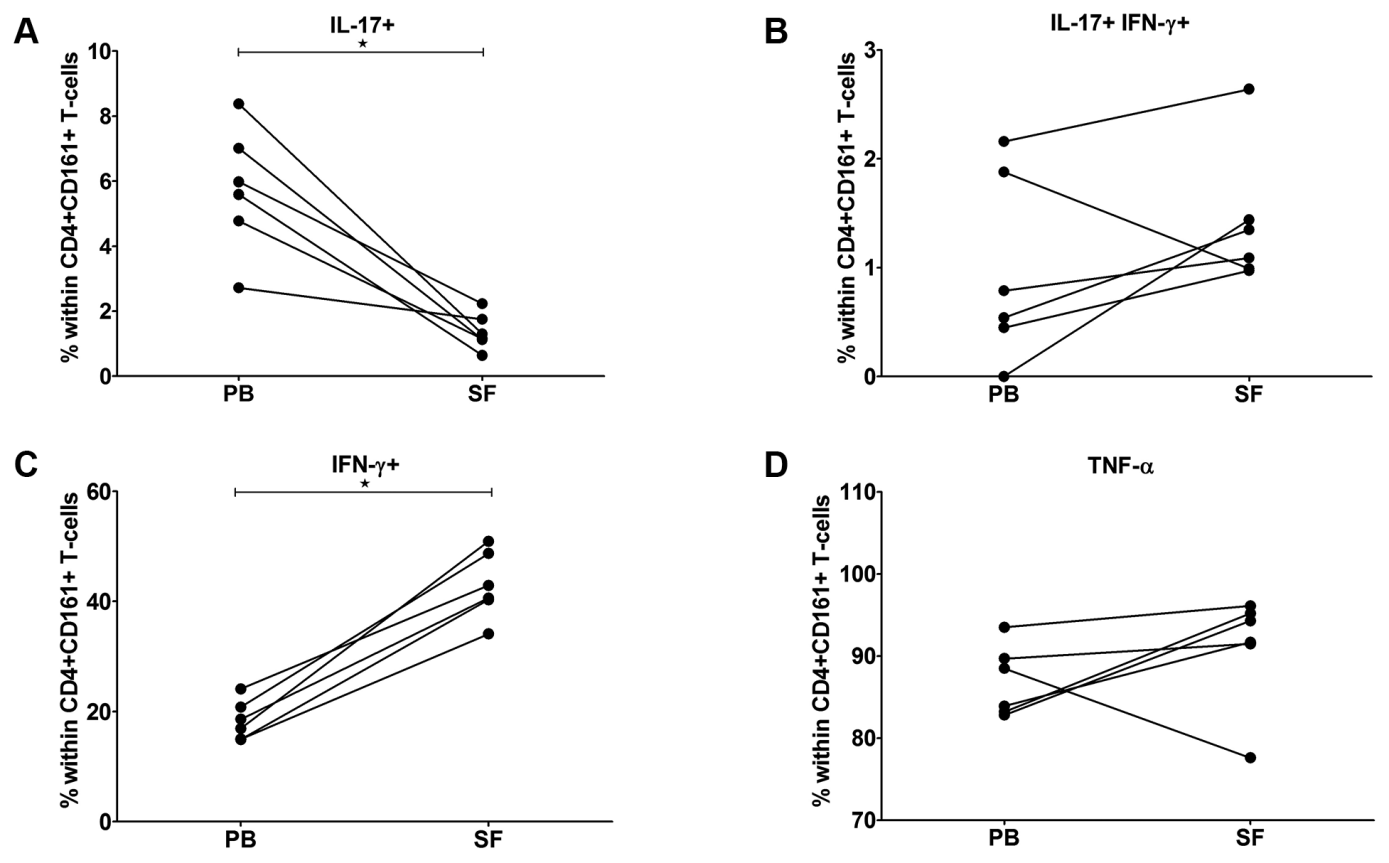
Synovial fluid CD4+CD161+ T-cells demonstrate a Th1 phenotype. Paired samples of PB and SF from late-stage RA patients (n=6) were stimulated using PMA/ionomycin in the presence of BFA. The frequency of CD4+CD161+ T lymphocytes producing (A) IL-17, (B) both IL-17 and IFN-γ (C) IFN-γ, or (D) TNF-α was assessed (Wilcoxon matched pairs test). Statistical significance is indicated as * p ≤ 0.05.

Since the CD4+CD161+ T-cells were detected at the level of the joints, we further investigated the Th17, Th17/Th1 and Th1 phenotypes and analyzed their cytokine producing potential in paired SF and PB samples from RA patients with late-stage disease. Interestingly, the percentage of IL-17-producing cells was significantly higher within the PB-derived CD4+CD161+ subset than the SF-derived subset (median value 5,1% vs. 1,6% within CD4+CD161+ in PB and SF, respectively, Figure 4A), while the capacity for production of both IFNγ and IL-17 was similar for PB- and SF- derived CD4+CD161+ T-cells (Figure 4B). Of note, a significant increase in the frequency of single IFNγ+ cells (non classical Th1) was seen in SF when compared to PB (median value 39,0% vs. 18,8%; Figure 4C). Expression of TNF-α was variable between CD4+CD161+ T-cells from PB and SF (Figure 4D). Thus, CD4+CD161+ T-cells in the joints of late-stage RA display a skewing towards a pro-inflammatory IFNγ signature. 

## Discussion

A better understanding of the immune events in the switch to clinical synovitis would open opportunities for prevention of RA development [4]. Thus, the definition of biomarkers discriminating between seropositive arthralgia and clinical synovitis is eagerly awaited. We report on profound changes in circulating precursor Th17 cells in SAP who are at risk of developing RA and in newly diagnosed patients with RA (before start of DMARD treatment). Whereas absolute numbers of CD4+CD161+ T-cells were found increased in the seropositive arthralgia group, a profound decrease of these cells was found to mark the early RA state. The decrease in the absolute number of CD4+CD161+ T-cells in early RA correlated inversely with CRP and with the SJC66. MTX treatment led to normalization of CD4+CD161+ T-cells and reduced disease activity. 

Our findings add to earlier reports implicating Th17 cells in the initiation phase of RA. Indeed, a cytokine environment favoring Th17 generation is an early event in RA pathogenesis [6]. Importantly, pre RA patients were found to show increased serum levels of IL-17 prior to the manifestation of clinical synovitis but these levels dropped significantly following the transition to RA [7]. The reported drop in systemic IL-17 levels is mirrored by our observation on the dynamics of Th17 precursor cells expressing CD161 in SAP (increase) and in clinical synovitis (decrease). Moreover, the inverse correlation with the SJC66 would suggest their homing to the joint. Indeed, CD4+CD161+ T cells were readily detected in ST from both newly diagnosed and late-stage RA patients. In addition, synovial fluid from late-stage RA was found to be enriched for CD4+CD161+ T-cells which is in line with the effector memory phenotype of these cells ( [21] and own observations). Migration of CD4+CD161+ T-cells to the joints is mechanistically explained by CCL20 induced migration. CCL20 expression in SF and ST has been reported previously to attract CCR6+ cells [24-26]. Notably, CCR6 expression is a feature of CD4+CD161+ T-cells. Both CD161 and CCR6 expression were found to be Th17 lineage transcription factor (RORC) dependent [16]. Transendothelial migration may be facilitated by CD161 mediated adhesion [23].

Interestingly, we found that the decrease in Th17 precursor cells was correlated with the SJC66 but not with the SJC28. The SJC66 includes ankles and feet, and thus provides a more comprehensive appreciation of joint involvement. This would be in line with earlier reports suggesting that the joints of the feet are important in very early RA [27]. 

In a prospective, longitudinal study, we demonstrated that peripheral CD4+CD161+ T-cell numbers normalize following regular MTX treatment. This may be explained by inhibition of migration due to MTX-mediated reduction of pro-inflammatory cytokines, chemokines and adhesion molecule expression in the joints [28,29]. It is currently not know if MTX affects CCL20 production, the primary chemokine for Th17 lineage cells. Our data thus reveal profound effects of MTX treatment on peripheral numbers of CD4+CD161+ cells and call for caution when interpreting data on cellular immune markers in patients receiving immune suppressive treatment.

In this study, we examined the dynamics of Th17 precursor T-cells in 3 unique cohorts of patients: in SAP who are at risk of developing RA, in newly diagnosed RA patients before and after start of MTX treatment and in late-stage RA. Previously, Miao et al reported on increased relative frequencies of IL-17 producing CD4+CD161+ T-cells that correlate with disease activity in RA [30]. This patient cohort had a mean disease duration of 3-4 years and most of these patients were treated with DMARDs. Thus, this study cohort can best be compared to our late-stage RA group on treatment. Although these authors did not report on absolute numbers of CD4+CD161+ cells, the reported percentages of IL-17 producing CD4+ CD161+ T-cells compare well with our data in late-stage RA (mean of 5% with ranges between 2-10%). 

In late-stage RA, synovial fluid CD4+ CD161+T-cells showed skewing towards the Th1 phenotype when compared to peripheral blood CD4+CD161+ T-cells. This is in line with previous data reporting on Th17 plasticity towards Th17/Th1 and Th1 cells [10,11]. The instability of the Th17 phenotype at the level of the joint may be explained by synovial fluid derived factors including IL-12 [11]. Alternatively, the plasticity of Th17 lineage cells at the level of the joint is explained by other mechanisms involving ligation of CD161 with naturally occurring ligands. The only confirmed endogenous ligand for CD161 is lectin-like transcript 1 (LLT1) [31-33]. LLT1 is expressed by activated antigen presenting cells and lymphocytes [34]. Interestingly, CD161 cross linking in vitro was shown to facilitate IFNγ production by T-cells [31,34]. Others reported on increased T-cell IL-17 production [35]. There is currently no data available on expression of LLT1 in RA. More studies are needed to assess if CD161-LLT1 ligation relays co-stimulatory signals and if this contributes to Th17 function and or Th1 skewing at the level of the joint in RA.

The search for biomarkers characterizing the transition to clinical synovitis is eagerly awaited and would present opportunities for prevention of RA. Also, candidate biomarkers would add to prediction models that are currently being developed [3,36]. Circulating Th17 lineage cells were increased in patients at risk for developing RA but decreased in newly diagnosed RA. MTX treatment led to normalization of circulating CD4+CD161+ T-cells. The decrease of CD4+CD161+ T-cells in early RA was associated with the SJC66. An improved mechanistic understanding of CD4+CD161+ T-cells in the switch to RA synovitis may ultimately provide novel treatment options.
